# The role of analytic direction in qualitative research

**DOI:** 10.1186/s12874-022-01546-4

**Published:** 2022-03-13

**Authors:** Joanna E. M. Sale

**Affiliations:** 1 Musculoskeletal Health and Outcomes Research, Li Ka Shing Knowledge Institute, St. Michael’s Hospital, Unity Health Toronto, 30 Bond Street, Toronto, Ontario M5B 1W8 Canada; 2 Institute of Health Policy, Management & Evaluation, University of Toronto, Health Sciences Building, 155 College Street, Suite 425, Toronto, Ontario M5T 3M6 Canada; 3 Department of Surgery, Faculty of Medicine, University of Toronto, 149 College Street, 5th Floor, Toronto, Ontario M5T 1P5 Canada

**Keywords:** Analytic direction, Qualitative research, Data analysis, Methodological rigour, Critical appraisal

## Abstract

**Background:**

The literature on qualitative data analysis mostly concerns analyses pertaining to an individual research question and the organization of data within that research question. Few authors have written about the entire qualitative dataset from which multiple and separate analyses could be conducted and reported. The concept of analytic direction is a strategy that can assist qualitative researchers in deciding which findings to highlight within a dataset. The objectives of this paper were to: 1) describe the importance of analytic direction in qualitative research, and 2) provide a working example of the concept of analytic direction.

**Methods:**

A qualitative dataset from one of the author’s research programs was selected for review. Ten potential analytic directions were identified after the initial phenomenological analysis was conducted. Three analytic directions based on the same coding template but different content areas of the data were further developed using phenomenological analysis (*n* = 2) and qualitative description (*n* = 1) and are the focus of this paper. Development and selection of these three analytic directions was determined partially relying on methodological criteria to promote rigour including a comprehensive examination of the data, the use of multiple analysts, direct quotations to support claims, negative case analysis, and reflexivity.

**Results:**

The three analytic directions addressed topics within the scope of the overall research question. Each analytic direction had its own central point or story line and each highlighted a different perspective or voice. The use of an inductive and deductive approach to analysis and how the role of theory was integrated varied in each analytic direction.

**Conclusions:**

The concept of analytic direction enables researchers to organize their qualitative datasets in order to tell different and unique “stories”. The concept relies upon, and promotes, the conduct of rigourous qualitative research.

## Background

Reports on data analysis in qualitative research are well documented. Procedural steps have been described [1–7] and authors have made distinctions between the concepts of coding, analysis, and interpretation [1, 2, 8, 9]. Authors have written about different researchers accessing different representations of a topic or phenomenon [2, 10] or multiple interpretations being applied to the same transcript [11]. The literature on data analysis mostly concerns analyses pertaining to an individual research question and the organization of data *within* that research question. Few authors have written about the entire qualitative dataset from which multiple and separate analyses could be conducted and reported.

The data collected by qualitative researchers can be voluminous and often surpass the data pertaining to objectives outlined in grant proposals. These data may be compelling but analyses of some data are often given lower priority if they do not align directly with the stated objectives.

There comes a point during data collection and analysis where qualitative researchers must choose “which story, of the many stories available to them in a data set, to tell” (p. 376) [12]. According to Arthur Frank, “[a] fter the methods, there has to be a story” (p. 431) [13]. “Stories” should have a central point or storyline [12]. The final report can be told from the perspective of different voices [12] and organized by time such as emphasizing key turning points and milestones in the sequence of events studied [12, 14] or by using other forms of representation such as metaphors [2, 12]. Theory can be central or more peripheral in the account [15]. The question remains, what “story”, or “stories”, do we tell?

### The concept of analytic direction

The concept of analytic direction is a strategy that can assist qualitative researchers in deciding which “stories” to highlight within a dataset. Sandelowski reports that researchers account for their data and then determine the different “paths” [1] or “analytic paths” [16] they can pursue. Others have proposed that decision-making throughout analysis implies analytic ideas at every stage of the coding process [8] and that researchers define for themselves what analytic issues are to be explored and what ideas are important [8]. Charmaz [17] reports that grounded theory researchers pursue more than one analytic direction by focusing on certain ideas first and then returning to the data to address an unfinished analysis in another area later. While the concept of analytic direction has been referenced, or alluded to, by these and other authors [1, 8, 16, 18, 19], operationalization of this concept is not well articulated. In this paper, the term analytic direction refers to a message developed by the researchers about the data that may or may not require further substantiation. An analytic direction can be presented as a single message or theme, and can stand alone or be supported by multiple sub-messages or sub-themes. Analytic directions can be developed during the coding process, in later stages of analysis, or possibly during analyses of new datasets. Relying on strategies to promote rigour can assist with the development, substantiation, and selection of analytic directions. If substantiated, each analytic direction could be the focus of an individual publication. The objectives of this paper were to: 1) describe the importance of analytic direction in qualitative research; and 2) provide a working example of the concept of analytic direction.

### Why analytic direction is important

The concept of analytic direction is important because it has implications for methodological rigour. We have an obligation to conduct methodological rigourous studies [20], especially when studies require primary data collection that involves a burden to participants [21]. The author proposes that methodological rigour is embedded within, and contributes to, the concept of analytic direction. Several strategies to promote rigour that are universal to many qualitative approaches, including phenomenology, are discussed. These strategies include, but are not limited to, a comprehensive examination of the data, the use of multiple analysts, direct quotations to support claims, negative case analysis, and reflexivity. It is important to support the quality of analytic directions so that researchers can then determine which analytic directions may or may not require further substantiation. The quality of the analytic direction will also assist in determining which directions may be selected for reporting.

### The relationship between analytic direction and methodological rigour

This paper focuses on the stage where data collection is considered to be complete and does not directly address how data collection, and methodological rigour related to data collection, contributes to the concept of analytic direction. The assumption is that data collection and analysis were conducted iteratively [22, 23] and that the team decided when data collection was complete, perhaps relying upon one of the various conceptualizations of saturation discussed by Saunders and colleagues [24]. A decision about saturation would not necessarily apply to any, or all, analytic directions being developed.

The author proposes that several strategies for promoting rigour assist with the development and selection of analytic directions. One aspect of methodological rigour is that authors carry out a comprehensive examination of their data [5, 25]. By thinking about, and engaging in, analytic direction, researchers are encouraged to attend to all of their data rather than attending only to data that interests them initially.

The use of multiple analysts promotes a comprehensive examination of the data [2, 26] and thus, contributes to the concept of analytic direction. Different viewpoints lead to an enrichment of the analysis and can lead to a conceptual clarification of the interpretations [2]. Multiple viewpoints can be used at the level of coding but also at the level of the larger team as data collection and analysis proceeds. Discussions about the novelty, clinical significance, and relevance [27] of the analytic directions may occur at this time and continue through to the writing of the respective manuscripts. Analytic directions are relevant if they add knowledge, or increase the confidence with which existing knowledge is regarded [28]. According to Malterud [26], engaging multiple researchers in a qualitative study strengthens the design of the study, not for the purpose of consensus or identical readings of the data but to supplement and contest each others’ statements.

The use of direct quotations to support the claims made about the analytic directions (and/or themes within) is another strategy to promote rigour [29]. Not only do quotations illustrate and clarify the results but they also demonstrate whether there is substantive evidence to support the analytic directions being proposed. In contrast, data that do not support the analytic directions (and/or themes within) should be accounted for and their exclusion justified when promoting methodological rigour [30]. Authors may refer to this as attending to negative cases [28] or deviant case analysis [25, 31]. This strategy promotes that “deviant cases” or “outliers” are not forced into categories or ignored but used instead to aid understanding or theory development [25]. For example, these cases may explain why the patterns developed from the data or the more normative behaviours are not always found in the researchers’ interpretations [25, 31].

Reflexivity is an essential component of methodological rigour [26]. Reflexivity has been described as “an attitude of attending systematically to the context of knowledge construction, especially to the effect of the researcher, at every step of the research process” [26] (p. 484). Being reflexive means being aware of your own position in producing partial knowledge [32]. The qualitative researcher acknowledges his or her personal influence on what that partial knowledge is (for example, the data collected are dependent on the interviewer’s questions and prompts). According to Eakin and Gladstone [33], knowing one’s standpoint helps one to recognize the forces that might drive certain interpretations and stifle other conceptualizations of the data. Knowledge production is also partial because it is not possible to report all interpretations of the data and therefore, the research team has to decide what to report. Researchers engaging in the concept of analytic direction are more likely to be reflexive about what they are, and are not, reporting from their datasets.

## Methods

### Rationale for the chosen example

The dataset chosen for this example was from a study where the author and her team identified 10 potential analytic directions based on a compilation of the memos and team discussions pertaining to analysis and interpretation of the data. The publications developed from this dataset reflected the selection of three analytic directions that focused on different content areas [34–36]. The same coding template was the foundation for the three publications and the timing of the reporting was ordered based on the author’s interests. The author chose the dataset as an example primarily because it was not heavily theory-laden and therefore accessible to novice qualitative researchers. The resulting publications have practical implications for clinical and health services research and the process of developing these publications could inform graduate students who are embarking on a qualitative program of research for their thesis work.

### Original research funded

The goal of the original research project was to reduce the burden of illness due to fracture through improved bone health investigation and treatment. Specifically, the aim was to examine what researchers could learn from members of a patient group. The study was approved by the Research Ethics Board at Unity Health Toronto (REB# 10–371). The study team consisted of scientists, clinicians, a policy maker, and a patient representative with expertise related to bone health. Informed by the Theory of Planned Behaviour [37, 38], the team set out to examine members of a patient group to ask them about their intentions and actions toward bone health diagnosis and treatment and their experiences with diagnostic tests and treatment recommendations. All individuals (*n* = 28) were 50+ years old and had sustained a fragility fracture. The overall project relied on a phenomenological approach conceptualized by Giorgi and Wertz [30, 39–41].

We developed a master coding template of 27 broad codes that were designed to organize the data with minimal reliance on theory. The coding template was revised four times as data collection and analysis proceeded. The codes were developed from a combination of inductive and deductive codes. More specifically, inductive codes were developed from topics discussed in the interviews. Other codes were pre-specified from the overall aim of the original funded study and from the domains of the Theory of Planned Behaviour.

### Development of analytic directions from the dataset

Qualitative researchers can use several strategies to develop analytic directions. The author started the organization process early in order to think about how best to maximize the data collected. Coding began after the first couple of interviews had been conducted; this is conventional advice for analysis in qualitative research [1, 2, 23, 42]. As soon as the coding process began, a document specific to analysis was created. Miles and colleagues have referred to this as “analytic memoing” [6]. This document is different from other documents in which the team discusses design features, decisions, and interview logistics related to the study. Analytic ideas were added to this document after coding and discussing each transcript. The author engaged two individuals in the coding/analysis process, as multiple analysts promote a comprehensive examination of the data [2, 26]. The author met regularly with members of the team during the process of data collection and analysis to discuss the data, interpretations of them, and different lines of inquiry. These discussions were recorded in the analysis document. Table 1 outlines the potential analytic directions considered for this paper. The 10 analytic directions were developed prior to publication of analytic direction #1. Some of these directions were posed as questions that required further analysis and substantiation. Tables were then created to help us to visualize patterns during analysis. As an example, for analytic direction #2, a table was created in which each participant was assigned a row and perceived messages from the various health care providers (for example, primary care providers and specialists) were placed in columns. Perceived messages were presented as quotations from participants. We examined the columns to compare perceived messages *across* provider groups for each participant and then examined the columns to compare the perceived messages *within* each provider group. For analytic direction #3, a table was created with each participant assigned a row and the domains of the Theory of Planned Behaviour assigned to columns. The table was populated with data in the form of quotations from each participant that we believed corresponded to each of the domains. Strategies such as matrices [5, 6] or thematic maps [42] can also be used to visualize developing patterns when presenting or organizing data.

**Table 1:** Potential analytic directions considered

Potential analytic directions	Notes about analytic direction
Strategies used by a “good” patient vs. a “patient advocate” appeared to differ^a^	• Participants talk about “doing as they are told”, following orders, being a good patient, even if they are experiencing side effects of the prescribed medication • Participants talk about doing what their doctor tells them but also trying to understand it and why, even if it means going to other health care providers for more information and for answers • Who are the participants who follow recommendations vs. those who do not – could this be influenced by patient characteristics and/or system characteristics? • What is the progression from being a “good” patient to being a patient advocate? • Some participants reported advocating for themselves until they found someone they trusted • Being a patient advocate is limited by the health care system (e.g. difficult to get a second opinion from health care provider)
Different motivations and routes to becoming a member of the patient group	• Some participants did not appear to join the patient group because they felt strongly about being a patient advocate • Some participants found the group on the internet while looking for information on bone health • Some participants were actively enrolled in the patient group through a fracture clinic or an osteoporosis program or through involvement in an Osteoporosis Chapter in their region • Being a member of a patient group may be just another source of information for individuals • Did the manner in which an individual became a member of the patient group reflect their experiences with bone health and recommendations for bone health?
There are many barriers in the health care system	• Some participants described challenges with getting a bone mineral density test (e.g. general practitioner as a potential barrier) • Health care system can be a barrier to accessing care (e.g. restricted access to specialists, the general practitioner not wanting to make a referral, limited specialists in participant’s geographic area) • How are participants able to get what they want/need (e.g. change in medications, referral to a specialist, information) despite system constraints?
Perceived messages by general practitioner and specialists to bone health as a health condition appear to vary^a^	• Perceived lack of seriousness of the condition or interest in the condition – participants not happy with their general practitioner either accepted this or sought care elsewhere (e.g. osteoporosis clinic, specialist) • Not feeling heard • Some participants requested a bone mineral density test and were denied getting the test or had to push for the test – in several cases, participants who pushed for the test reported compromised bone health on test results • Some participants requested a referral to a specialist but were not given a referral • Receiving bone health care sometimes attributed to luck (e.g. a medical student prompted the further investigation) • Care related to bone health by a general practitioner vs. a specialist not always the same • Both general practitioners and specialists did not appear to recommend non-pharmacological strategies to manage bone health, including supplements and exercise
Patients talk differently about compromised bone health vs. being at risk for future fracture	• Health care providers need to articulate the importance of bone health as well as the importance of reducing one’s fracture risk • Discussions about bone health differed from discussions about fracture risk • Sometimes difficult to understand whether participants connected their previous fractures with bone health • Some participants reported that the term “osteoporosis” was more frightening than being “high risk” for future fracture • Participants reported that they should hear about bone health from within the medical system and not outside it (e.g. from Osteoporosis Canada)
Several factors appeared to influence participants’ perceptions of bone active medication	• Some questioned whether the medication was working or not • Reported belief that doing something is better than doing nothing • Some participants expressed a desire or hope that they might be able to stop taking the medication in future • A few participants refused to “do as told” because they did not like taking medication in general – this was not specific to bone active medication • Age and the presence or absence of other health conditions appeared to influence one’s attitude to starting, or continuing to take, bone active medications
Participants appeared to have a favourable view of bone active medication	• Most participants did not have an issue or complaint about the idea of starting, and/or taking, bone active medication prescribed • There appeared to be a lot of participants who had switched bone active medications several times • Participants were willing and interested in trying new medications as they became available • Participants appeared to be very aware of new bone active medications on the market • Participants switched medications due to experiencing side effects • Participants appeared to be pro-medication to the point where their idea of “good” care was getting a prescription for the best medications on the market. This attitude seemed to persist despite individuals re-fracturing and/or experiencing side effects while taking the medication
Choice to take medication appeared to influence participants’ engagement in non-pharmacological strategies	• How does taking medication influence participants’ perceptions of what else they can do with respect to managing bone health? • Some participants perceived they had more of a role in their bone health if they chose not to take bone active medication prescribed
What is the relationship between the Theory of Planned Behaviour and pharmacological and non-pharmacological treatment?^a^	• Initially, the Theory of Planned Behaviour did not appear to be very useful in explaining medication initiation and/or use • Participants do not speak in the language of behaviour change models – the domains are difficult to match to participants’ language • Difficult to separate non-pharmacological strategies for an in-depth analysis of the Theory of Planned Behaviour domains • Difficult to code intentions retrospectively
What is the relationship between the Theory of Planned Behaviour and bone mineral density testing?	• The domains of the Theory of Planned Behaviour do not appear to factor into participants’ decision to go for a bone mineral density test. • Participants do not appear to have issues with going for a bone mineral density test – they do not appear to need to be convinced to go for the test • Difficult to code intentions retrospectively

^a^Analytic direction pursued for further analysis and selected

### Selection of the three analytic directions

The number of analytic directions selected likely depends on circumstances including the quality of the data, the quality of the analysis, and available resources. The research team considered the multiple analytic directions, discussing their relevance [27], novelty, and clinical significance and also the interests of the team in order to incorporate the perspectives of the different stakeholders. It was important to the author that the content of each analytic direction was bounded in that it did not overlap with the content of the other analytic directions. For example, analytic directions #2 and #3 discuss the potential influence of others in participants’ lives. However, analytic direction #2 focused on health care providers while analytic direction #3 focused on family members, friends, and colleagues of participants and specifically excluded health care providers from the analysis based on the Theory of Planned Behaviour domain “subjective norm”. In narrowing down the list of analytic directions, the author ensured there were sufficient data (quotations) to support the claims. Cases that did not fit the general results were acknowledged in order to justify their exclusion or explain why they did not fit. For example, in analytic direction #3, we examined instances where the data did not appear to fit with the Theory of Planned Behaviour and explained what happened in these instances where the model did not appear to be predictive of intentions.

The master coding template was important as it assisted with the organization of evidence for each analytic direction. The master coding template also assisted the team with the creation of tables for each analytic direction discussed. Table 2 demonstrates the relationship between the master coding template and the three selected analytic directions.

**Table 2:** Master coding template and relationship to the three analytic directions selected (codes are not mutually exclusive)

Code	Examples of code	Analytic Direction
Ambiguity	Any confusion about bone health diagnosis, bone mineral density testing, treatment, bone health recommendations	
Attitude to bone mineral density testing	Theory of Planned Behaviour domain “attitude”	Analytic direction #3
Attitude to bone health treatment	Theory of Planned Behaviour domain “attitude”. Includes motivation to manage bone health	Analytic direction #3
Barriers/facilitators	Includes barriers/facilitators to bone health management, bone mineral density testing, and treatment	
Bone health treatment	Source for Theory of Planned Behaviour domain “perceived behavioural control”. Treatment includes bone health medication and supplements	Analytic direction #3
Bone mineral density test results	Any discussion about the results of the test	
Bone mineral density testing experience	Booking the test, going to the test facility, having the test	
Canadian Osteoporosis Patient Network (COPN) involvement	Includes any discussion about the patient group or involvement with Osteoporosis Canada (who supports COPN)	
Fear of re-fracture	Includes perceived likelihood of having another fracture	
Fracture clinic	Includes all events and interactions that occurred within the fracture clinic such as speaking to the orthopaedic surgeon	Analytic direction #2
Fractures	Everything said about the fracture experience, including the emergency room experience, previous fractures, healing process	
General practitioner	Any discussion about participant’s general practitioner and interactions with the general practitioner	Analytic direction #1 Analytic direction #2
Health care system	Includes discussion about continuity of care, referrals within the health care system, the transfer of participant’s medical information between health care providers	Analytic direction #1 Analytic direction #2
Intentions	Theory of Planned Behaviour domain “intentions”. Includes intentions regarding bone mineral density testing, bone health treatment, having a specific plan	Analytic direction #3
Learn from participant	Overall aim of funded grant. Includes what messages health care providers and researchers should give to patients	
Linking fracture to bone health	Includes participants connecting their fracture to bone health or not	
Other conditions and medications	Other conditions that participants have, including acute and chronic conditions	
Other health care providers	Includes other specialists and health care providers, including heart specialist, physiotherapists, chiropractors, dieticians	Analytic direction #2
Other bone health strategies	Includes non-pharmacological strategies (other than supplement use) for bone health recommended to participants or carried out by participants such as diet, exercise, being careful, avoiding falls, seeking out information	
Pain	Includes discussions about current pain, related to the fracture and not related to the fracture	
Patient centred care	Includes descriptions of participants seeking information, demanding care, having a pro-active role in their health, or the absence of this behaviour	Analytic direction #1
Perception of bone health status	Participants’ interpretation of the status of their bone health	
Perceptions of general health	Includes talk about aging and attitude to aging	
Recommendations for bone mineral testing received	Any recommendations received by health care providers for bone mineral density testing	Analytic direction #1 Analytic direction #2
Recommendations for treatment received	Any recommendations received by health care providers (orthopaedic surgeon, physiotherapist, general practitioner) for bone health treatment	Analytic direction #1 Analytic direction #2
Social influence	Source for Theory of Planned Behaviour domain “subjective norm”. Includes pressure to perform or not perform bone mineral density testing and bone health treatment. Includes other sources of information such as the internet, television shows, magazines, parent with osteoporosis, lectures attended	Analytic direction #3
Specialist for bone health	Information from or interactions with specialists for bone health such as a rheumatologist, endocrinologist, internal medicine, osteoporosis clinic attended	Analytic direction #1 Analytic direction #2

The impetus for analytic direction #1 [34] was based on an assumption held by the author as she was working on the research proposal. Her expectation was that members of a patient group would be patient advocates who were experts in navigating for care. She was interested in what patients could learn from members of this patient group. The analytic direction for the paper came from surprise, and subsequent disappointment, that those assumptions were not supported by the data and that members of the patient group did not all appear to be advocates and experts in navigating for care. One commonality that defined the patient group was that members appeared to be in favour of taking prescribed medication.

Analytic direction #1 included elements of both inductive and deductive analysis in that codes were developed for the master coding template from the data (inductive) but the author’s expectations also influenced how those codes were combined and how the team interpreted the data (deductive). Drawing from the literature, the term “advocacy” was equated with the theoretical concept of “effective” or “activated” consumer [43, 44]. The code “effective consumer” did not exist in the original master template, partly because we preferred to not apply theoretical labels prematurely to the data. Based on the coding template, we drew from six codes to create a table about “effective consumer” behaviours (see Table 2). Participants were then coded along a continuum between what was referred to as “few effective consumer behaviours” (patients who followed orders with minimal involvement in their care and demonstrating the least amount of advocacy) to “many effective consumer behaviours” (individuals demonstrating significant involvement in their care, those who demanded diagnostic testing and requested specific medications).

Analytic direction #2 [35] was developed concurrently with analytic direction #1. The role of theory was minimal in analytic direction #2 and perhaps implicit in the methodology of phenomenology which focuses on individuals’ experiences [23, 39]. The impetus for analytic direction #2 was our proposal that messages from health care providers might determine individuals’ strategies or behaviours that were the focus of analytic direction #1. The analysis was more inductive than that of analytic direction #1 as the team had no pre-contemplated plan to examine how messages from health care providers might determine individuals’ behaviours. In conducting the analysis, the team wondered whether conflicts about what individuals did with the recommendations they received (their actions) appeared to be due to messages perceived across, and within, health care provider groups. Health care providers discussed in the interviews included clinic staff, primary care providers, specialists, nurses, physiotherapists, and chiropractors.

For analytic direction #2, we used seven of the codes in the master coding template (see Table 2). Five of these seven codes were also used in analytic direction #1 but for very different reasons and drawing from different data within these codes. We were interested in individuals’ understanding or interpretation of recommendations by health care providers, not how individuals interacted with health care providers or what they did with information received from health care providers. In other words, we were interested in the *meaning* of what health care providers reportedly said to participants and not what participants *did* with that information.

The publication for analytic direction #3 [36] was written 3 years after that for analytic direction #1. This was the author’s least preferred paper, despite the Theory of Planned Behaviour being the theoretical framework guiding the original funded research. Analytic direction #3 involved a primarily deductive analysis where the Theory of Planned Behaviour guided the coding and analysis. Because of the restrictions of forcing exploratory data from open-ended questions into pre-defined domains, the author selected a qualitative description approach for the research design.

Contrary to memos and reflexive notes documented by the author about the potential value of this analysis and whether the team had learned anything about the application of the Theory of Planned Behaviour in the context of our study, the pursuit of analytic direction #3 became an interesting methodological exercise for a number of reasons. We collected data on several behaviours including receiving diagnostic tests, taking supplements, exercising, attending falls prevention classes, and initiating medication. The author believed that one particular behaviour had to be selected for analysis which entailed examining the data for each of the behaviours in depth. The author chose to focus on medication initiation and/or medication use because of a longstanding interest in medication use. Also, there was sufficient data to substantiate the Theory of Planned Behaviour domains in relation to medication initiation and/or medication use. The Theory of Planned Behaviour did not appear to be particularly relevant to intentions to attend a bone mineral density test and there did not appear to be sufficient data to support any one of the non-pharmacological treatment strategies mentioned. The team also had to make decisions about what counted as “perceived behavioural control”, “subjective norms”, and “attitudes” which were the three domains of the Theory of Planned Behaviour [37, 38]. In particular, participants’ discussions about medication side effects were problematic to conceptualize in reference to these domains. The team decided to code “*experiences* with side effects” as “perceived behavioural control” but “*anticipated* side effects” as an “attitude”.

For analytic direction #3, the team drew from five codes, three of which were pre-specified prior to analyzing the interviews and meant to capture the domains of the Theory of Planned Behaviour. The code “attitude to BMD testing” and “attitude to bone health treatment” were existing codes based on the Theory of Planned Behaviour. The code “subjective norm” was not part of the coding template because the team believed it was too specific. We instead examined the code “social influence” which captured a broader array of information about peers such as family members and friends. Similarly, “perceived behavioural control” was not part of the coding template because we found it too specific. Information for this domain was taken from another code labelled “bone health treatment” which captured data pertaining to participants’ medications, including past behaviour with medication and how difficult it was, or not, to take the medication. The code “intentions” was an existing code.

## Results

The three selected analytic directions varied in how the team used an inductive and deductive approach to analysis [15, 45] and how the role of theory was integrated (“central” vs. more “peripheral”) [15]. Each publication was within the scope of the overall research goal or question. As proposed by Agee [46], this overall question offered the potential for more specific questions during analysis. Finally, each publication had its own central point [12] and highlighted a different perspective or voice [12].

The following is a summary of the three analytic directions labelled with the first few words of the titles of each publication (see Table 3).

**Table 3:** Three analytic directions selected

Analytic Direction #1	Title	Strategies used by a patient group
	Objective/purpose	To examine the experiences and behaviours with bone health management post-fracture among members of an existing national patient group
	Emphasis on inductive versus deductive analysis	Inductive and deductive
	Analytic approach	Phenomenology
	Key messages	More than half of the participants described effective consumer behaviours, including making requests of bone health care providers for referrals to bone specialists, bone mineral density tests, and prescription medication. These behaviours could be translated into skill sets and incorporated in post-fracture interventions.
Analytic Direction #2	Title	Perceived messages about bone health
	Objective/purpose	To determine how members of a national bone health patient group perceive the messages received from various healthcare providers regarding bone health following a fracture
	Emphasis on inductive versus deductive analysis	Inductive
	Analytic approach	Phenomenology
	Key messages	Most participants perceived that their specialist was more interested than their primary care provider in bone health and took the time to discuss issues with them. There were many instances where perceived messages within and across various healthcare providers were inconsistent.
Analytic Direction #3	Title	Theory of Planned Behaviour explains intentions to use medication
	Objective/purpose	To determine if the Theory of Planned Behaviour explains patients’ intentions to use, or continue using, bone active medication after a fracture.
	Emphasis on inductive versus deductive analysis	Deductive
	Analytic approach	Qualitative description
	Key messages	The Theory of Planned Behaviour appeared to be predictive of intentions to use medication in approximately three-quarters of participants. In the majority of participants where the Theory of Planned Behaviour did not appear to be predictive, a positive attitude toward the medication was the most important domain in determining intentions.

### Analytic direction #1 (Strategies used by a patient group; inductive and deductive-driven)

In this publication, we examined the strategies described by three groups of individuals: individuals demonstrating few effective consumer behaviours, individuals demonstrating many effective consumer behaviours, and individuals demonstrating both types of behaviours. We discussed how the continuum was contrary to our expectations of what behaviours members of a patient group would exhibit. Having acknowledged this finding, we reported that more than half of the participants described effective consumer behaviours including making requests of health care providers for referral to specialists, bone mineral density tests, and prescription medications. Our overall message was that members of a patient group described a range of effective consumer behaviours that could be incorporated as skill sets in post-fracture interventions.

### Analytic direction #2 (Perceived messages about bone health; inductive-driven)

In this publication, we described the perceived messages *across* the different provider groups and then the perceived messages *within* each provider group. We reported that participants perceived that specialists were more interested in their bone health than general practitioners and that very few messages about bone health were perceived from other health care providers. We also reported that perceived messages about one’s bone health and recommendations for management across provider groups were inconsistent (for example, with regard to medication initiation). The message for analytic direction #2 was that patients perceived inconsistent messages within, and across, various healthcare providers, suggesting a need to raise awareness of bone health management guidelines to providers.

### Analytic direction #3 (Theory of Planned Behaviour explains intentions to use medication; deductive-driven)

In this publication, we described the data in each domain of the Theory of Planned Behaviour and the apparent relationship between these domains and participants’ intentions with regard to medication use. Our message was that the Theory of Planned Behaviour appeared to be predictive of intentions to take prescribed medication in approximately three-quarters of participants and when it was not predictive, a positive attitude to medication was the most important domain in determining participants’ intentions.

## Discussion

This working example of analytic direction resulted in three publications highlighting distinct "stories”. The publications differed in a number of ways. Each publication had its own central point or story line [12]. The role of theory [15] was minimal in analytic direction #2 but was more central in analytic directions #1 and #3 with the concept of “effective” or “activated” consumer and the Theory of Planned Behaviour dominating the analyses, respectively. Acknowledging that the authentic voices of participants may always be manufactured by the authorial account [32, 47], all papers were written from the perspective of “I” or “we”. However, we focused on *participants* at the forefront for analytic direction #1 and we focused on participants’ perceptions of their *providers’* voices for analytic direction #2. For analytic direction #3, the voice of the *research team* dominated as we struggled with methodological decisions. It is proposed that the voice of the *model* (Theory of Planned Behaviour) also dominated in analytic direction #3.

One implication related to analytic direction is that the research team may need to modify elements of the original research design to better suit the analytic direction selected. If such a modification is made, the team should ensure theoretical consistency in how the methods and methodologies are integrated [48, 49]. For example, Crotty [49] proposes that theoretical consistency is needed between methods, methodology, theoretical perspective, and epistemology because these four elements inform one another. Similarly, Carter and Little [48] argue that consistency between methods, methodology, and epistemology contribute to the rigour of a qualitative study. Authors should demonstrate that elements of their theoretical perspectives and research design are compatible if they are applying another methodological approach to the data. Carter and Little [48] suggest that methodologies can be combined or altered if the researcher retains a coherent epistemological position and justifies the choices made. In the funded grant, a phenomenological program of research was proposed and the data were collected through in-depth interviews conducted from a phenomenological perspective. Analytic direction #3 was not purely consistent with a phenomenological approach because of the restriction to force exploratory data into domains of a theoretical framework and so we pursued this analytic direction with a different approach (qualitative description). As pointed out by Sandelowski [50], using phenomenology and qualitative description in this way is not to be confused with misuses of methods or techniques. Unlike quantitative research, qualitative research is not produced from any “pure” use of a method, but from the use of methods that are variously textured, toned, and hued [50]. According to Sandelowski [50], qualitative description can be used in conjunction with phenomenological research in a number of ways. For example, phenomenological analyses can be applied to qualitative descriptive studies [50]. However, the pursuit of other approaches to analysis, such as grounded theory or a participatory action approach, might lead to epistemological tensions if the original study design and data collection was guided by a phenomenological approach. Future discussion about the concept of analytic direction when considering theoretical and methodological positions that differ epistemologically from the original design and conduct of the study is needed.

There are a number of other implications related to the concept of analytic direction. Practically, it is advised that researchers start to think about analytic directions early so that they are aware of the potential analytic directions being developed as soon as data collection and analysis begin. By thinking about the “larger picture” at this early stage in the research, the team is better equipped to make the most of the data collected. Having said this, one will likely never use the entire dataset. As researchers, we rarely have sufficient funds or personnel to pursue all analytic directions. Data are often set aside because researchers are eager to analyze data collected for new projects or pressured to seek future funding opportunities. Analytic directions that are not pursued can be transferred to student projects. Alternatively, it is possible to draw on a sub-set of the transcripts/observations to carry out a secondary analysis. The author has developed subsequent analytic directions that span across studies and draw from a subset of transcripts for several secondary analyses [51–53]. Analytic directions can also contribute to ideas for new grant proposals that enable the researcher to generate more data on analytic directions that need further substantiation and further exploration.

This paper demonstrates some guidance about how to bound each analytic direction. Bounding the analytic direction is necessary so one does not re-use the data or produce multiple, yet quite similar, papers on the same topic. Researchers are encouraged to be open and transparent and acknowledge related publications so reviewers and other audiences reading the work are able to determine for themselves that the analyses are different.

There are ethical considerations in developing an analytic direction or framing the analytic direction in a way that might be different or supplementary to the original design. It is not always feasible to obtain subsequent consent from participants for use of the data if this use differs from that of the original goal of the study. As a result, analytic directions pursued should be within the scope of the approved research ethics application. One strategy is to keep the study goal or aim broad in the research ethics submission so that it encompasses many topics that might be discussed during data collection. Another consideration is to not prematurely close a research ethics application because researchers may be able to use the data for a secondary analysis at a later date.

This paper makes novel contributions to qualitative research methodology by demonstrating how the process of analytic direction works, by operationalizing the concept and providing an example, and by describing the connection between analytic direction and rigour. This paper further contributes to the advancement of rigour by demonstrating how the development and selection of analytic directions relies on several strategies to promote rigour, such as a comprehensive examination of the data, the use of multiple analysts, providing quotations to support claims made, checking for negative cases, and reflexivity.

## Conclusions

In conclusion, the concept of analytic direction enables researchers to organize their qualitative datasets in order to tell different and unique “stories”. The concept relies upon, and promotes, the conduct of rigourous qualitative research. As with all elements of qualitative analysis, researchers are encouraged to think about the role of analytic direction as soon as data collection commences.

## Data Availability

The datasets generated and/or analysed during the current study are not publicly available due to participants not consenting to having their data deposited in a public dataset but are available from the corresponding author on reasonable request.
